# The Difficulties of Occipito-Posterior Presentations

**Published:** 1909-06-19

**Authors:** 


### Obstetrics.

### THE DIFFICULTIES OF OCCIPITO-POSTERIOR PRESENTATIONS.

Of all obstetric difficulties, those encountered when the vertex presents in the occipito-posterior positions are the commonest. They are responsible too for a very considerable fœtal mortality, and for a large proportion of the maternal injuries and morbidity in child-birth. These facts are by no means universally recognised, chiefly because posterior vertex positions are so often undiagnosed before birth and unrecognised after it. Many a patient is said to have contracted pelvis on the strength of one or more difficult labours, the real causes of which were occipito-posterior presentations.

The difficulties in connection with these presentations are chiefly the non-appreciation of the frequency with which these positions of the vertex occur, the difficulties of diagnosis, the difficulties of the mechanism of natural delivery, and finally the proper treatment of them when diagnosed. The third vertex presentation or right occipito-posterior occurs, according to Eden, in 20 per cent. of the vertex presentations, and the left occipito-posterior in 1 per cent. only. The latter figure is in accordance with most authorities, but the probability is that it is too low, and that it is really nearer to 5 per cent. In any case, nearly a quarter of all vertex presentations have the occiput posterior with regard to the pelvis. The reason why anterior positions of the occiput are more common than posterior is usually said to be because the fœtus accommodates itself more readily thus, owing to the large size of the occiput as compared with the frontal end of the head—the anterior parts of the pelvis being roomier than the posterior.

Difficulties of diagnosis occur because vaginal examinations are even now relied upon, instead of abdominal palpation. It is often a matter of great difficulty to recognise the position of the sutures and fontanelles of the fœtal head when felt per vaginam. On the other hand, it is quite easy to diagnose posterior positions from the abdomen if the usual methods are adopted. The mere fact that the arms and legs of the fœtus can be easily felt through the abdominal wall, and the prominence of the back cannot be recognised, is generally sufficient to indicate an occipito-posterior position. This, coupled with the recognition of one shoulder to the right or left of the mid line, makes absolutely certain the presence of an occipito-posterior position. The mechanism of delivery depends upon the presence or absence of flexion of the head on the trunk, and on a recognition of the anatomical shape of the important parts of the pelvic floor. All vertex presentations are flexed at first, but when the head enters the pelvis during the last weeks of pregnancy, the posterior occipital presentations are apt to have this flexion undone and slight extension substituted. The reason for this lies in the want of room in the posterior parts of the pelvis, which consequently prevents the large occipital end of the head descending as easily as the frontal end. It is a useful law to remember that the part of the head which lies in front always descends easiest, and this applies equally to the breech presentations. Thus the head becomes deflexed to a greater or lesser extent, and the result may be that it sinks through the pelvis with neither the occiput nor the forehead anterior. That one or the other end of the head should be in advance is necessary for the rotation movement to occur normally, for whatever part is in advance will turn to the front. Hence it follows that if the forehead and occiput lie horizontally level they will impinge on the pelvic floor of their respective ideas at the same moment. They will then each try to rotate to the front, with the result that neither does so, and labour comes to a standstill in spite of strong uterine contractions. Fortunately, in most occipito-posterior cases the amount of deflexion which occurs is not great and is not sufficient to make the forehead the lowest part, or, what is more to the point, is not sufficient to prevent the occiput from being the lowest part. Consequently rotation takes place normally, and the occiput rotates to the front. If the forehead becomes the lowest part owing to excessive deflexion, then it naturally touches the pelvic floor first, rotates to the front, and a persistent occipito-posterior results. It is, of course, particularly the cases in which labour comes to a standstill because neither end of the head rotates, in which long delay and great difficulties arise, chiefly because the cause of these difficulties is unrecognised.

If these facts concerning the mechanism are grasped, treatment follows as a matter of common sense. Clearly we must have accurate diagnosis, and then we must supplement the mechanism by artificial aids. The simplest procedure is to flex the head by pulling on the occiput with fingers and holding it in this position during uterine contractions. This will not often succeed by itself unless the contractions are very powerful. It is much more often necessary to rotate the head with the whole hand introduced into the vagina. The head can be grasped with the whole hand, and the occiput can be artificially flexed and rotated around the right side of the pelvis in third positions and the left side in fourth positions. This manœuvre is assisted by pushing the anterior shoulder across the mid-line of the abdomen away from the occiput with the operator's external hand. It is not always possible to push the shoulder across, especially if all the liquor amnii has drained away. In such a case it is good treatment to rotate the head, disregard the twisting of the neck which occurs, and then put on forceps to prevent the head returning to its original position, and to deliver as an occipito-anterior. The method which has perforce to be adopted when the occiput is persistently posterior and lies in the hollow of the sacrum must depend upon the size of the child and the strength of the uterine contractions. In some cases the mother may deliver herself, but more often the head will have to be pulled out in this position with the forceps.

